# Association Between Metabolic Clusters and Microbial Age in High-Risk Populations for Diabetes and Their Potential Impact on Cardiovascular Disease Risk: Cross-Sectional Observational Study

**DOI:** 10.2196/73119

**Published:** 2026-05-26

**Authors:** Lu Xinlin, Hongli Gu, Ren Li, Xianjun Mao, Can Chen

**Affiliations:** 1 Department of Cardiovascular Medicine, Jinan University, Guangzhou, China; 2 Department of Cardiovascular Medicine, Chenzhou First People’s Hospital, Hunan, China; 3 Department of Ultrasound Medicine, The First People's Hospital of Chenzhou, Chenzhou, China; 4 Department of Cardiovascular Medicine, The First People's Hospital of Chenzhou, Chenzhou, China; 5 Department of Cardiovascular Medicine, Affiliated Hospital of Guangdong Medical University, No.57, Renmin Avenue South, Xiashan District, Zhanjiang, Guangdong Province, 524001, China, 86 0759-2369336

**Keywords:** metabolic multimorbidity, microbial age, gut microbiota, cardiovascular disease risk, diabetes

## Abstract

**Background:**

Metabolic multimorbidity is prevalent in high-risk populations for diabetes and is linked to cardiovascular disease (CVD) and gut microbiota composition. The relationship between metabolic clusters (MCs), microbial age (MA), and metabolic markers remains poorly understood.

**Objective:**

This study aimed to investigate the characteristics of MCs and MA in high-risk diabetic populations, focusing on their associations with gut microbiota, metabolic dysregulation, and CVD risk.

**Methods:**

Using data from the NIH Integrative Human Microbiome Project, we performed metabolomic and microbiomic analyses. K-means clustering identified MCs, and redundancy analysis examined the relationship between metabolic variables and microbiota. A random forest (RF) model predicted MA and CVD risk, while the linear discriminant analysis effect size identified microbial species associated with MCs and MA. Co-occurrence network analysis explored microbial interactions.

**Results:**

We included 103 high-risk individuals (56/103, 54.4% female, mean age 50.6, SD 54.6 years). In total, 3 MCs were identified: MC1 (high glucose or blood urea nitrogen), MC2 (relatively healthy), and MC3 (lipid dysregulation). Age explained 3% of gut microbiota variation (*R*^2^=0.03; *P*=.006). The RF model predicting microbial age showed a strong correlation with chronological age in training data (ρ=0.97, root mean square error=3.33; *P*<.001) and moderate correlation in test data (ρ=0.35; *P*<.001). High microbial age was associated with elevated lipid markers (low-density lipoprotein and triglycerides; *P*<.001) and higher cardiovascular risk. The RF model for CVD risk prediction achieved excellent discrimination (area under the curve=0.95 for the low-risk and 0.95 for the high-risk groups).

**Conclusions:**

This study highlights the relationship between MCs, MA, and gut microbiota, providing insights for early intervention and personalized treatment strategies for diabetes and related metabolic disorders.

## Introduction

According to the International Diabetes Federation (IDF), approximately 463 million adults worldwide had diabetes in 2019, and this number is expected to continue rising over the coming decades [1]. Diabetes is a metabolic disorder characterized by chronic hyperglycemia, typically accompanied by abnormalities in insulin secretion or action, or both. This persistent high blood glucose not only disrupts glucose metabolism but is also closely linked to severe complications such as cardiovascular disease (CVD), kidney disease, and neuropathy [2,3]. Early in the disease, patients with diabetes often have concurrent metabolic disorders, such as hypertension, obesity, and dyslipidemia, which collectively form metabolic clusters (MCs) [4,5]. These metabolic abnormalities interact and exacerbate the condition, further increasing the CVD risk in patients with diabetes [6,7]. Thus, early identification of high-risk populations with diabetes is critical for personalized treatment and precision medicine.

In recent years, the gut microbiota, as a crucial microbial ecosystem in the human body, has been found to play a significant role in host metabolism, immunity, and neurological functions [8,9]. Extensive research has shown that the imbalance of the gut microbiota is closely related to metabolic disorders in diabetes and its complications. The gut microbiota influences the host’s glucose, lipid metabolism, and inflammatory responses through metabolic products such as short-chain fatty acids (SCFAs) [10,11]. However, the mechanisms underlying the interaction between the gut microbiota and the host’s metabolic functions remain incompletely understood. Nevertheless, growing evidence suggests that changes in the gut microbiota’s composition are not only associated with the pathogenesis of diabetes but may also contribute to the comorbidity of other metabolic diseases, such as obesity, fatty liver disease, and hypertension, providing a biological basis for the formation of MCs [12,13].

The health of the gut microbiota is closely linked to the biological age of the host. The composition and function of the microbiota can reflect the host’s biological age, a concept known as “microbial age” (MA) [14,15]. MA is a composite indicator inferred from the analysis of the gut microbiota’s composition, metabolic products, and other factors. It is closely associated with the host’s health status, metabolic function, and inflammation levels [16,17]. Research has shown that, with increasing host age, the gut microbiota undergoes natural aging, characterized by reduced microbial diversity and increased specific pathogens [18,19]. Therefore, as a potential biomarker, MA may provide new insights for early prediction and personalized intervention of metabolic diseases.

The potential application of MA in high-risk populations with diabetes has attracted growing attention. MCs and dysbiosis typically characterize these populations, and the interaction of these multiple factors may exacerbate disease progression and the onset of complications [20,21]. Existing studies have indicated that MA is associated with the onset of various diseases, including metabolic disorders, CVDs, and obesity. However, research on MA in high-risk diabetic populations remains limited [22,23]. CVD, one of the most common complications of diabetes, has become a leading cause of death in high-risk populations with diabetes [2,24]. Therefore, understanding the relationship between MCs, MA, and CVD is of significant clinical importance for developing personalized intervention strategies.

This study aims to explore the characteristics and interrelationships between MCs and MA in high-risk populations with diabetes through metabolomics and microbiome analysis and to reveal the potential impact of these factors on CVD risk. We will focus on analyzing the relationships between different metabolic subtypes and the gut microbiota in high-risk populations with diabetes, investigating the interactions between metabolic disorders and changes in the microbiota. Through systematic data analysis, we will uncover the potential role of MA in metabolic disorders and CVD risk and further identify key microbial species that may exhibit significant differences across different metabolic subtypes and MA groups, thereby influencing the host’s metabolic health and disease risk. In addition, we will explore the synergistic interactions within the gut microbiota, further revealing how microbial communities contribute to the development of metabolic diseases through various mechanisms. This study will not only deepen our understanding of the complex relationship between metabolism and microbiota in high-risk populations with diabetes but also provide new scientific evidence and clinical perspectives for the early diagnosis, personalized treatment, and intervention of diabetes and related metabolic diseases.

## Methods

### Ethical Considerations

This study was a secondary analysis of publicly available, deidentified data from the NIH Integrative Human Microbiome Project (iHMP). The original studies were approved by the relevant institutional review boards, and all participants provided written informed consent at the time of data collection. As this analysis used only anonymized data and involved no direct contact with participants, additional ethical approval was not required and the study was considered exempt from further review. Participant privacy and confidentiality were fully protected. No compensation was provided for this secondary analysis.

### Data Acquisition

This study was based on data from the NIH Integrative Human Microbiome Project (iHMP), focusing on individuals at high risk for diabetes. A total of 103 individuals were included in the study cohort, contributing longitudinal samples collected during infection (n=79) and healthy states (n=429) across multiple time points. To avoid the confounding effects of acute illness on metabolic and gut microbiome profiles, only samples from the healthy phase were selected for the main analyses, including metabolic subtype classification, MA prediction, and CVD risk evaluation. This selection ensured that results reflected stable metabolic characteristics and minimized biases introduced by transient infection.

The sample types consist of fecal samples (16S ribosomal RNA microbiome), clinical data (BMI, age), and laboratory metabolic data (glycated hemoglobin, albumin, blood urea nitrogen [BUN], calcium, cholesterol, high-density lipoprotein cholesterol, chloride, creatinine, estimated glomerular filtration rate, glucose, high-density lipoprotein [HDL], high-sensitivity C-reactive protein, insulin, potassium, low-density lipoprotein [LDL], sodium, non–high-density lipoprotein, triglycerides, and total protein).

Subjects from both the iHMP and subprojects, such as exercise, were included. Detailed demographic and metabolic characteristics are listed in Table S1 in Multimedia Appendix 1. Females made up approximately 54.4% (56/103) of the overall sample; the majority were White, with a minority being Black or mixed race. The average age was 50.6 (SD 54.6) years, and the median BMI was 26.7 (IQR 19.4‐39.1) kg/m^2^. Some participants also had recorded steady-state plasma glucose levels and insulin resistance or sensitivity status, which were used for subsequent stratified analyses. This diverse cohort covered a range of metabolic statuses and population characteristics, providing a robust basis for investigating the relationship between metabolic subtypes and MA.

### Construction of MCs

To characterize MCs, we applied an unsupervised K-means clustering algorithm to the metabolic data from the healthy phase, ensuring that short-term fluctuations due to infection did not affect the clustering outcomes. This approach improved the biological interpretability and robustness of the identified subtypes. Initially, metabolic data from individuals at high risk for diabetes were collected, standardized, and tested for various clustering numbers (K-values). The optimal number of clusters was determined by calculating the Davies-Bouldin index and using the NbClust function to compute the Kullback-Leibler index. Based on these results, K=3 was selected as the optimal number of clusters, and the data were partitioned into 3 clusters (MC1, MC2, and MC3) using the k-means algorithm. Principal component analysis was applied to visualize the clustering results and validate the distribution of each cluster. Additionally, heatmaps were generated to illustrate the distribution of variables across clusters. Data analysis and visualization were primarily performed using the R packages NbClust, clusterSim, and factoextra.

### Redundancy Analysis

Redundancy analysis (RDA) was used to explore the relationship between the gut microbiome and metabolic variables. The RDA was performed using the RDA function from the R package Vegan, applied to both microbiome and metabolic data. Subsequently, the ANOVA function (with 999 permutations) was used to calculate each variable’s contribution to the explained variance and identify significant results. The results were converted to percentages and displayed using bar plots to visually represent the explained variance for each metabolic variable.

### Distance-Based Redundancy Analysis

To analyze the relationship between different MCs and gut microbiome composition, we performed distance-based redundancy analysis (dbRDA) using the R packages “ggrepel,” “dplyr,” and “vegan.” First, the Bray-Curtis distance was used to compute a distance matrix for the microbiome data, with age and multiple metabolic indicators as independent variables. A dbRDA model was then constructed. To assess the differences in the microbiome composition between MCs, permutational multivariate ANOVA was applied, with 999 permutations to calculate the *P* values.

### Random Forest Analysis

To explore the relationship between microbiome characteristics and host age, we used a random forest (RF) model with 5-fold cross-validation, implemented in the R packages “randomForest,” “caret,” and “tidyr,” to predict MA. The microbiome data were combined with the corresponding metadata and split into 70% training and 30% test sets. The model parameters were optimized through 5-fold cross-validation, and the RF model was trained on the training set to predict MA. Model performance was evaluated on both the training and test sets using Spearman correlation and root mean square error (RMSE). The results were visualized with a correlation scatter plot, illustrating the relationship between actual and predicted ages. Additionally, uniform manifold approximation and projection dimensionality reduction was applied to display the distribution of different MCs and low microbial age (LMA) and high microbial age (HMA) in the reduced dimensional space, providing visual support for subsequent analyses. The model was constructed using 500 decision trees (ntree=500), with 5-fold cross-validation used to tune the mtry parameter (ranging from 2 to 6). A random seed of 123 was set to ensure reproducibility. All model training, cross-validation, and performance evaluation were conducted in R using standard packages, including randomForest, caret, and pROC.

### Linear Discriminant Analysis Effect Size

To investigate the distribution of significantly different microbial species across different MCs and MA, linear discriminant analysis effect size (LEfSe) was used, with a threshold for linear discriminant analysis (LDA) effect size set at 2. The microbiome data were first standardized to ensure comparability in statistical analysis. LEfSe was then used to test the significance of microbial species in different MCs, identifying those with significant differences across groups. Nonparametric Kruskal-Wallis tests were conducted to assess species abundance differences between groups, followed by LDA to calculate the effect size for each significant species. Species with an LDA score greater than 2 were selected to quantify the intergroup differences.

### Co-Occurrence Network Analysis

To reveal the co-occurrence patterns between different microbial populations or genes, we performed correlation analysis using the “propr” package in R (R Core Team). First, the microbial abundance data were standardized to minimize the impact of differences in data magnitude on the analysis. The “propr” package was then used to calculate the phi coefficient (φ) between populations, as this coefficient effectively handles high-dimensional zero-inflated data and reduces the impact of sparsity on correlation assessments. Based on the results, we selected significantly correlated pairs using a predefined significance threshold (φ ≥ 0.3; *P* value threshold=.05) to construct the co-occurrence network. Network nodes represent microbial populations or genes, and edges indicate significant correlations. Subsequently, modularity analysis was conducted to identify functionally related or synergistic microbial communities, and the modularity index was calculated to assess the quality of network clustering. The network structure was optimized using the Fruchterman-Reingold layout algorithm, with community modules indicated by different colors.

### Framingham Risk Assessment

We used the Framingham risk score model to assess the study participants’ CVD risk. Data, including age, sex, blood pressure, total cholesterol, low-density lipoprotein cholesterol, high-density lipoprotein cholesterol, and diabetes status, were collected. Using the Framingham risk assessment formula, we calculated each participant’s probability of developing CVD within the next 10 years. Based on the risk scores, participants were classified into low, medium, and high-risk groups, enabling an analysis of the relationship between different risk levels, gut microbiome composition, and MCs.

### RF Model for Predicting CVD Risk

This study used an RF model to predict CVD risk, optimizing model performance through hyperparameter tuning. The dataset was split into 70% training and 30% test sets. We set the random seed to 123 to ensure reproducibility and used 5-fold cross-validation to optimize the mtry parameter (ranging from 2 to 6), training 500 decision trees. The model evaluation included the generation of a confusion matrix to assess classification accuracy and specificity, along with extracting feature importance scores to identify key metabolic indicators. To evaluate the RF model’s performance in predicting CVD risk, we used the pROC package to generate ROC curves for each risk category (low, medium, and high) and calculated the corresponding area under the curve (AUC) values.

## Results

### Construction of MCs and Analysis of Gender Differences and Metabolic Characteristics

Diabetes is a metabolic disorder characterized by prolonged hyperglycemia, typically resulting from insufficient insulin secretion or impaired insulin action [25]. Increasing evidence suggests that the gut microbiome plays a crucial role in the onset and progression of diabetes. The gut microbiome, consisting of diverse microorganisms, including bacteria, fungi, and viruses, is essential for maintaining human health, digestion, absorption, and immune regulation. Recent studies have shown that the gut microbiome in individuals with diabetes exhibits distinct structural and functional characteristics compared to healthy individuals [26].

**Figure 1.**
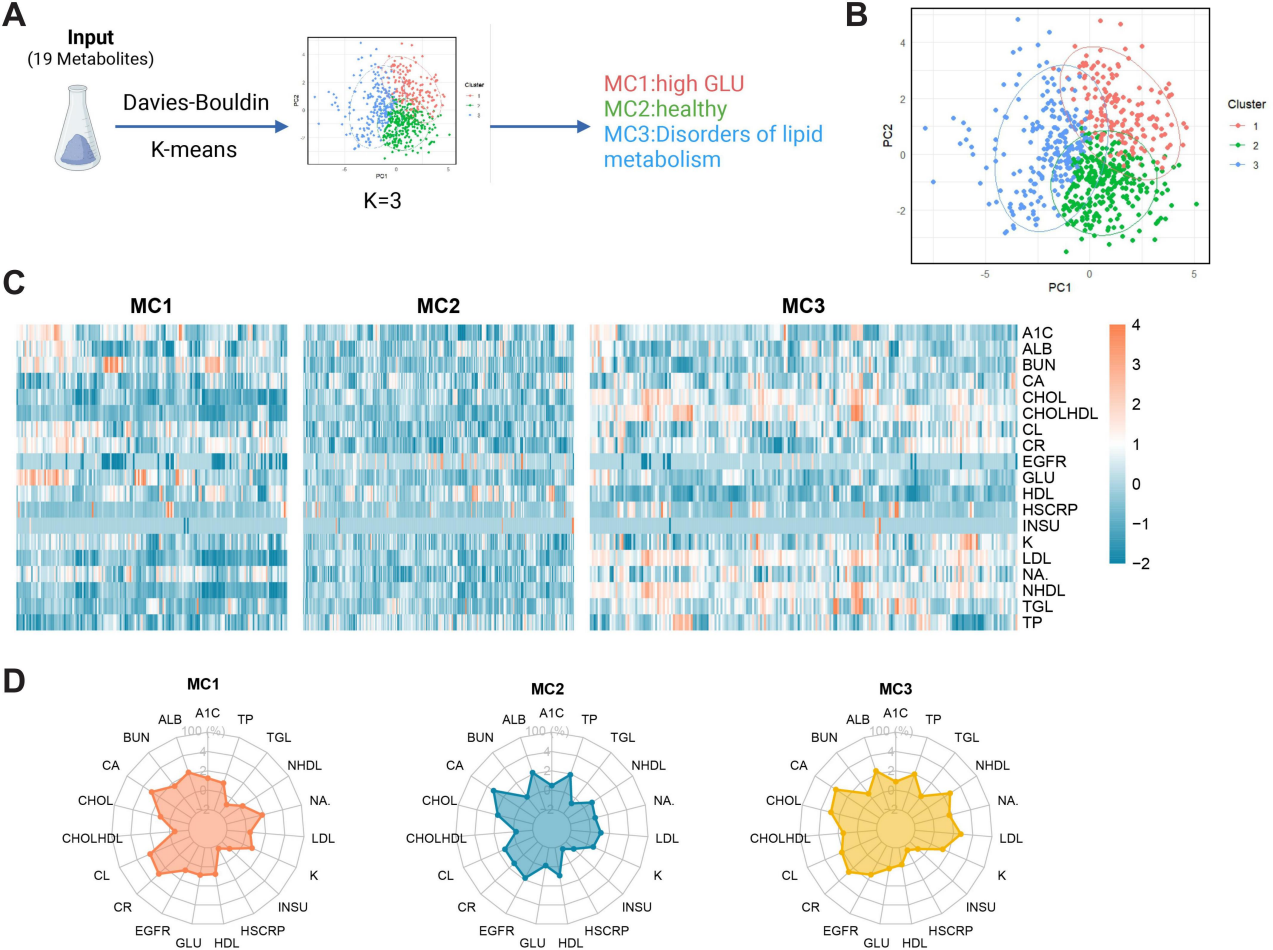
Metabolic subphenotype classification and characterization based on k-means clustering. (A) Study workflow: based on 19 metabolic variables, k-means clustering was applied, with the optimal number of clusters determined to be 3 using the Davies-Bouldin index and Kullback-Leibler index. In total, 3 metabolic clusters were identified: MC1 (high glucose), MC2 (healthy metabolic profile), and MC3 (lipid metabolism dysregulation). (B) Principal component analysis of MCs: the distribution of the three clusters is shown in the PC1 and PC2 space, with colors representing MC1 (red), MC2 (green), and MC3 (blue). (C) Distribution of standardized metabolic variable values across different metabolic clusters, with color indicating the relative level of each metabolic variable (orange for higher, blue for lower). (D) Characteristic distribution of 19 metabolic indicators across different metabolic clusters. A1C: hemoglobin A_1c_; ALB: albumin; BUN: blood urea nitrogen; CA: calcium; CHOL: cholesterol; CHOLHDL: high-density lipoprotein cholesterol; CL: chloride; CR: creatinine; EGFR: estimated glomerular filtration rate; GLU: glucose; HDL: high-density lipoprotein; HSCRP: high-sensitivity C-reactive protein; INSU: insulin; K: potassium; LDL: low-density lipoprotein; NA: sodium; NHDL: non–high-density lipoprotein cholesterol; TGL: triglyceride; TP: total protein.

This study used an unsupervised k-means clustering algorithm to analyze 19 precisely measured metabolic variables and classified 103 participants (including data from both infection and healthy phases) into different metabolic MCs (Figure 1A). The optimal number of clusters was determined to be 3 based on the Davies-Bouldin index and the Kullback-Leibler index. Principal component analysis showed that the 3 clusters exhibited a certain degree of separation in the PC1 and PC2 space, although some sample overlap was observed in certain regions (Figure 1B). We believe that this overlap reflects the continuous spectrum nature of metabolic states in high-risk populations for diabetes, rather than strictly discrete pathological classifications. This may better resemble clinical reality, suggesting that some individuals are in transitional stages of metabolic states. These borderline samples between metabolic phenotypes may exhibit greater plasticity in disease progression and, therefore, warrant special attention in individualized risk assessment and intervention strategies. Moreover, since this study was based on a single cohort, the current clustering results have not yet been externally validated. In the future, we plan to incorporate independent, multicenter population data to further assess the stability and generalizability of the metabolic classification.

Each MC exhibited distinct metabolic subphenotypes: MC1 was characterized by elevated glucose and BUN levels; MC2 represented a relatively healthy metabolic profile; and MC3 was characterized by elevated cholesterol, high-density lipoprotein cholesterol, potassium, LDL, triglyceride, and total protein levels. MC1 may indicate glucose metabolism and kidney function issues, whereas MC3 may be associated with lipid metabolism disorders and potential cardiovascular risk. In contrast, MC2 displayed a more ideal metabolic state, potentially reflecting metabolic homeostasis or better overall health (Figure 1C and D).

**Figure 2.**
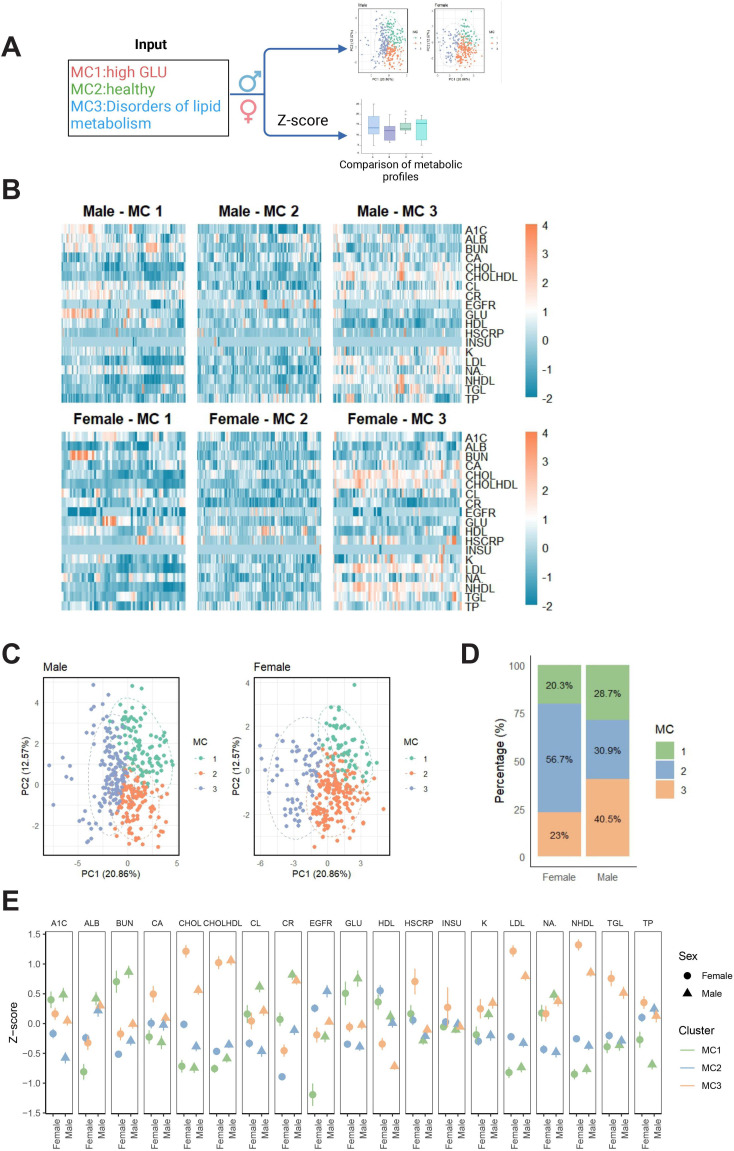
Metabolic characteristics analysis based on gender and MCs. (A) Study workflow: metabolic clusters were identified using k-means clustering based on the values of 19 metabolites. *z*-score normalization was applied to analyze gender differences in metabolic characteristics across the metabolic clusters. (B) Distribution of metabolic variables in different genders and metabolic clusters (MC1, MC2, and MC3), with color representing the *z*-score of metabolic levels (orange for higher, blue for lower). (C) Distribution of metabolic clusters in the PC1 and PC2 space for males and females, with different colors indicating different metabolic clusters. (D) Proportional distribution of males and females in each metabolic cluster, with females showing a significantly higher proportion in MC2, while males have a higher proportion in MC3. (E) Comparison of *z*-scores of metabolic indicators between males and females across different MCs. A1C: hemoglobin A_1c_; ALB: albumin; BUN: blood urea nitrogen; CA: calcium; CHOL: cholesterol; CHOLHDL: high-density lipoprotein cholesterol; CL: chloride; CR: creatinine; EGFR: estimated glomerular filtration rate; GLU: glucose; HDL: high-density lipoprotein; HSCRP: high-sensitivity C-reactive protein; INSU: insulin; K: potassium; LDL: low-density lipoprotein; NA: sodium; NHDL: non–high-density lipoprotein cholesterol; TGL: triglyceride; TP: total protein.

To explore metabolic differences between genders and across MCs, we used k-means clustering analysis, principal component analysis, and *z*-score normalization methods (Figure 2A). The results showed that, overall, there were no significant gender differences in metabolic characteristics. However, women tended to exhibit higher metabolic levels for certain metabolites, particularly those related to carbohydrate and nitrogen metabolism (eg, BUN and glucose; Figure 2B–C). Moreover, there were notable differences in the distribution of MCs between males and females. While MC2 had the highest proportion in both groups, the proportion of females in MC2 (57%) was significantly higher than that of males (41%). Conversely, a higher proportion of males (29%) was found in MC3 compared to females (23%; Figure 2D). The gender differences in MC1 were minimal, showing typical metabolic characteristics. In MC2, there were small metabolic differences between genders, though the overall metabolic levels were low, possibly indicating a suboptimal health status. In the MC3 group, a clear sex difference was observed, with female participants showing more pronounced metabolic features, such as higher *z*-scores for cholesterol, high-sensitivity C-reactive protein, LDL, and non–high-density lipoprotein compared to males (Figure 2E). This may suggest a greater degree of lipid metabolism disorders and chronic inflammation among females in this group. This phenomenon could be closely related to the regulatory role of sex hormones, especially estrogen, in lipid metabolism. It may also be influenced by gender-differentiated factors such as lifestyle, social role distribution, and dietary behavior. Studies have shown that women, particularly during the perimenopausal period, are at increased risk of lipid metabolic imbalance due to hormonal changes, and the gut microbiota may also be involved in metabolic regulation through estrogen-related pathways [27]. Therefore, gender is a critical factor in both the composition of MCs and the mechanisms underlying MA. Future studies should consider incorporating variables such as hormone levels, menstrual cycle status, and dietary habits to better elucidate their roles in the differentiation of metabolic phenotypes.

### Associations Between Host Age, MCs, and Gut Microbiome

**Figure 3.**
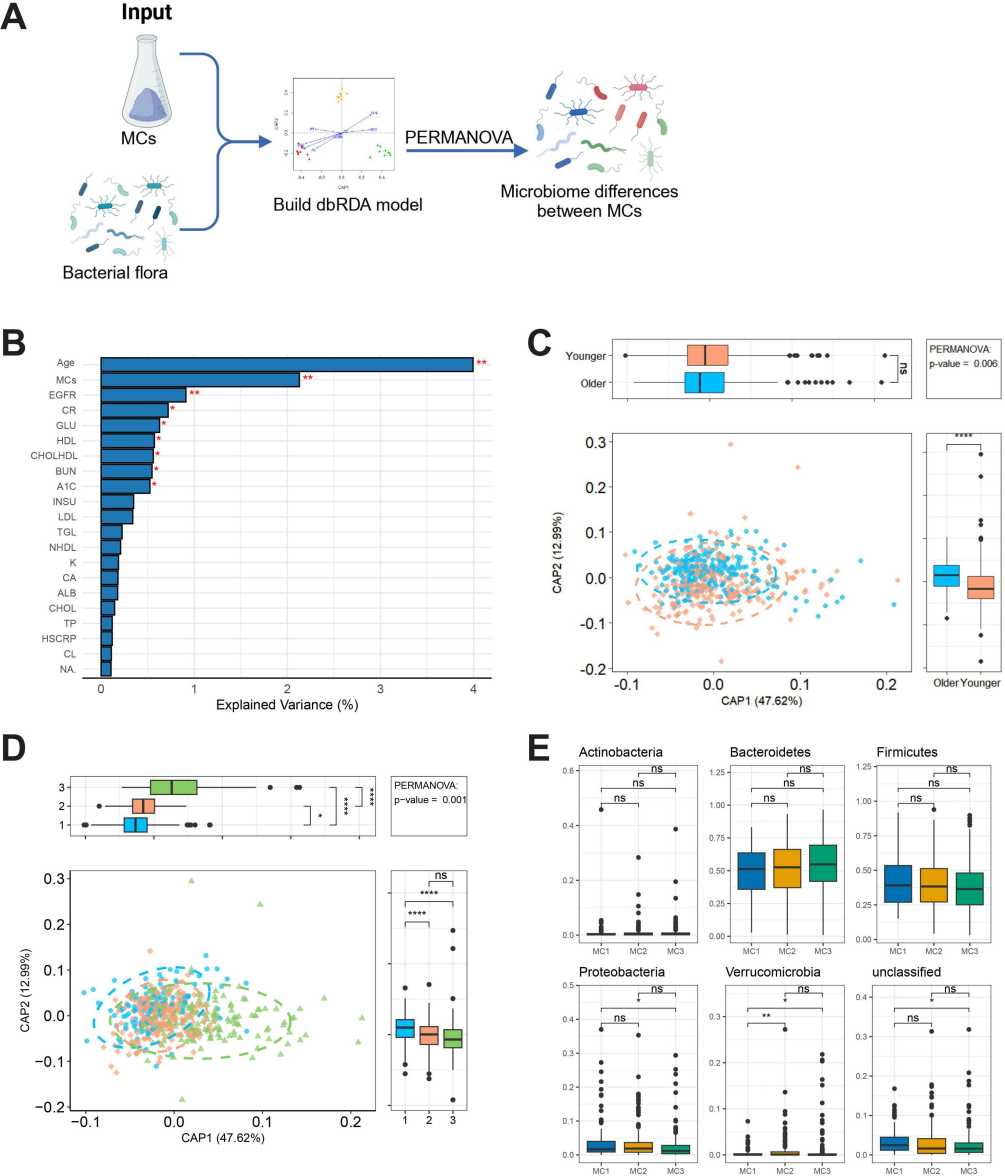
Association analysis of metabolic clusters, age, and gut microbiota composition. (A) Study workflow: gut microbiota data were obtained through 16S ribosomal RNA sequencing, and a dbRDA model was constructed, incorporating metabolic clusters and age. Permutational multivariate ANOVA was used to identify significant differences in microbiota composition. (B) Redundancy analysis of variable contributions: The explanatory contributions of age, metabolic clusters, and metabolic variables to gut microbiota changes are displayed, with age (*R*²=3%) being the strongest covariate. (C) Age-related differences in gut microbiota composition: distance-based redundancy analysis axes CAP1 and CAP2 explained 61% of the compositional variation, with significant differences observed between younger and older groups in microbiota structure (permutational multivariate ANOVA; *P*=.006). (D) Gut microbiota differences among metabolic clusters: distance-based redundancy analysis revealed significant compositional differences between MC1, MC2, and MC3 (permutational multivariate ANOVA; *P*.001), with CAP1 explaining 48% of the variation. (E) Key differences in gut microbiota phylum-level abundances between different metabolic clusters: Kruskal-Wallis and Dunn tests identified significant abundance differences in Proteobacteria and Verrucomicrobia phyla across metabolic clusters. A1C: hemoglobin A_1c_; ALB: albumin; BUN: blood urea nitrogen; CA: calcium; CHOL: cholesterol; CHOLHDL: high-density lipoprotein cholesterol; CL: chloride; CR: creatinine; dbRDA: distance-based redundancy analysis; EGFR: estimated glomerular filtration rate; GLU: glucose; HDL: high-density lipoprotein; HSCRP: high-sensitivity C-reactive protein; INSU: insulin; K: potassium; LDL: low-density lipoprotein; NA: sodium; NHDL: non–high-density lipoprotein cholesterol; TGL: triglyceride; TP: total protein; PERMANOVA: permutational multivariate ANOVA.

We obtained 16S ribosomal RNA data from gut samples to investigate the relationship between the gut microbiome and host and environmental factors (permutational multivariate ANOVA; Figure 3A). Redundancy analysis was performed to explore how 19 metabolic variables, age, and MCs contribute to the variation in microbiome composition. Notably, age emerged as the strongest host-associated variable affecting microbiome composition (*R*²=3%). After accounting for age and other covariates, MCs and most MC-related variables also showed significant contributions. To further explore the relationship between age and gut microbiome composition, participants were divided into 2 groups: a younger group (<60 years) and an older group (≥60 years; Figure 3B). The dbRDA revealed significant associations between age, MCs, and the gut microbiome composition, with the first 2 axes explaining 61% of the total constrained variation. Statistically significant differences in microbial community structure were observed between the younger and older groups (*P*value threshold=05; Figure 3C). Additionally, significant differences in microbiome composition were found between the different MCs (MC1, MC2, and MC3; *P*<.05). The microbiome profiles of MC1, MC2, and MC3 showed distinct differences, reflecting the impact of metabolic status on gut microbiome structure (Figure 3D). Kruskal-Wallis and Dunn tests were used to identify gut microbes with significant abundance differences between MCs. The results revealed that the 2 most prominent phyla were Proteobacteria and Verrucomicrobia. The relative abundance of Proteobacteria showed only slight differences across MC1, MC2, and MC3, with significant variation observed only between MC1 and MC3 (*P* value threshold=.05). In contrast, Verrucomicrobia exhibited more pronounced differences between groups, with significant relative abundance differences found particularly between MC1 and MC2, as well as between MC1 and MC3 (*P* value threshold=.05; Figure 3E).

### Construction and Validation of MA

**Figure 4.**
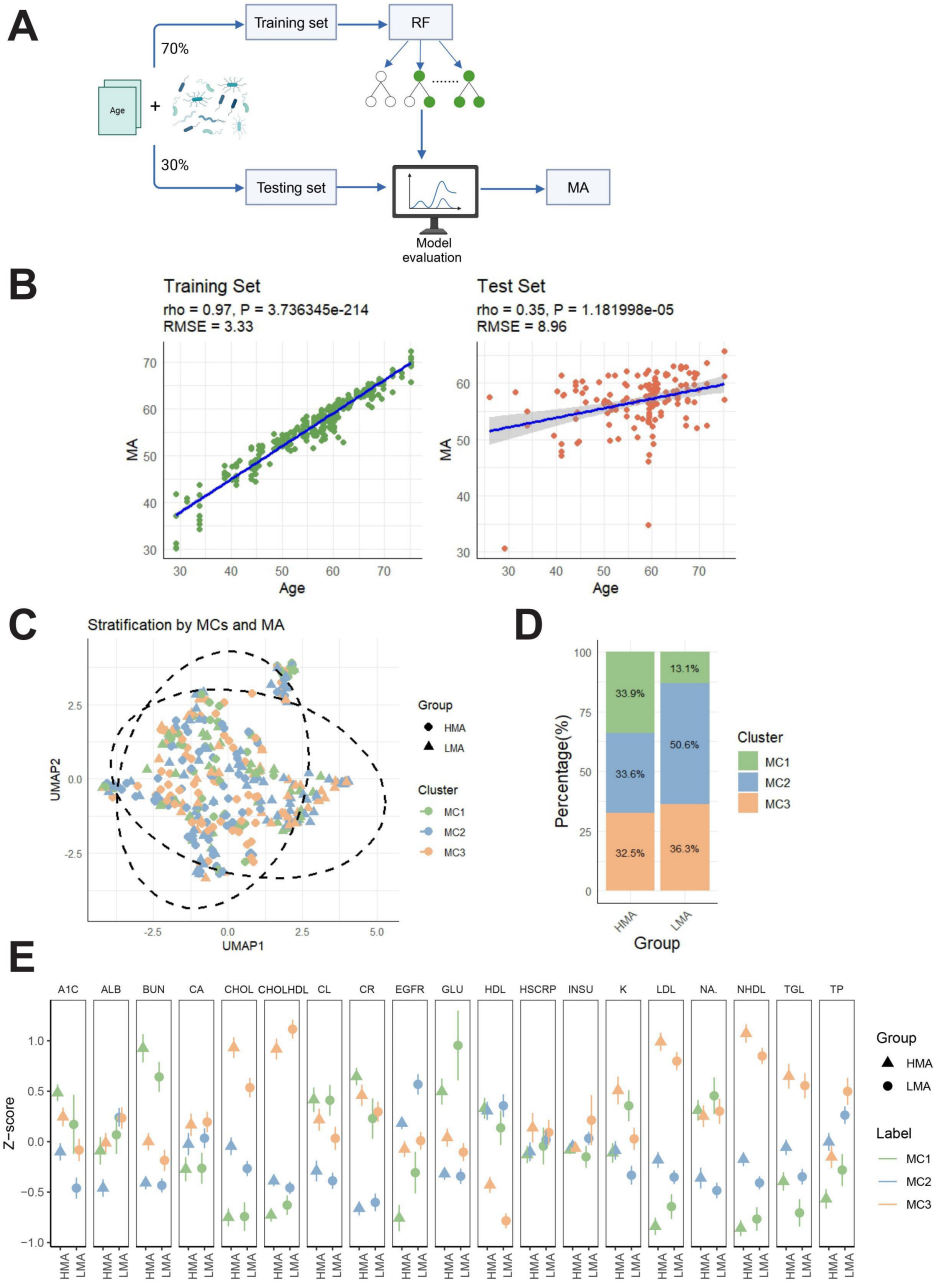
MA prediction model and metabolic feature analysis. (A) Study workflow: A random forest model was constructed to predict microbial age using age and significantly different microbial taxa. Data were divided into a training set (70%) and a test set (30%), with performance evaluation conducted. (B) Scatter plots of actual age versus predicted microbial age in the training and test sets. Root mean square error represents the average prediction error, and rho indicates the strength of monotonic correlation between predicted and actual values; (C) Uniform manifold approximation and projection dimensionality reduction showing the distribution of LMA and HMA across different metabolic cluster groups. (D) Proportion of MC1-MC3 in each microbial age group. (E) z-score differences in metabolic indicators (eg, low-density lipoprotein, triglycerides, non–high-density lipoprotein) between microbial age groups. Colors represent microbial age classifications, and symbol size indicates sample quantity. A1C: hemoglobin A_1c_; ALB: albumin; BUN: blood urea nitrogen; CA: calcium; CHOL: cholesterol; CHOLHDL: high-density lipoprotein cholesterol; CL: chloride; CR: creatinine; EGFR: estimated glomerular filtration rate; GLU: glucose; HDL: high-density lipoprotein; HMA: high microbial age; HSCRP: high-sensitivity C-reactive protein; INSU: insulin; K: potassium; LDL: low-density lipoprotein; LMA: low microbial age; NA: sodium; NHDL: non–high-density lipoprotein; RMSE: root mean square error; TGL: triglyceride; TP: total protein; UMAP: uniform manifold approximation and projection.

We conducted a Kruskal-Wallis test to analyze the differences in microbial species abundance between different age groups (younger group:<60 years, older group:≥60 years) and identified microbial species that showed significant differences between these age groups. Based on 20 significantly different species, we constructed a five-fold cross-validation RF model to predict MA (Figure 4A). The model results showed a high correlation in the training set (ρ=0.97; *P* value threshold=.01) and a low RMSE of 3.33, indicating a strong relationship between age and MA within the training data. The model performed well on this dataset. In the test set, the model still showed a significant correlation (ρ=0.35; *P*=1.18e-05), suggesting an association between age and MA. Although the model demonstrated high predictive correlation in the training set (ρ=0.97; RMSE=3.33), its performance in the test set was relatively limited (ρ=0.35; RMSE=8.96), suggesting that the model’s generalizability to unseen samples requires improvement (Figure 4B). In addition, the current MA model was built based solely on 20 differential microbial genera and did not incorporate microbial functions or metabolic products, which may oversimplify the complex microbiome features associated with age. Future work could reference related studies [28] to integrate metabolomics, functional genomics, or host-microbiome interaction data in order to develop a more comprehensive and robust MA prediction model and enhance its utility as a biomarker. After stratifying participants into LMA and HMA groups, we found that the microbial composition differences between the MA groups were significantly greater than the differences observed between the three MCs (Figure 4C). Additionally, the distribution of MCs was similar between the 2 MA groups. Notably, MC2 was the dominant cluster in the LMA group (51%), suggesting that MC2, representing a relatively healthy metabolic profile, predominates in individuals with LMA (Figure 4D). Next, we examined the differences in metabolic levels (measured in *z*-scores) between the LMA and HMA groups across different MCs (MC1, MC2, and MC3). The results showed that cholesterol, high-density lipoprotein cholesterol, LDL, non–high-density lipoprotein, and triglycerides were higher in MC3 than in MC1 and MC2, and higher in the HMA group compared to the LMA group. These findings suggest that MC3 is closely associated with lipid metabolism abnormalities, particularly in the HMA group, which exhibited higher levels of lipid metabolism dysregulation. This may indicate that higher MA is linked to disturbances in lipid metabolism (Figure 4E).

### Microbial Community Differences in MCs and MA

**Figure 5.**
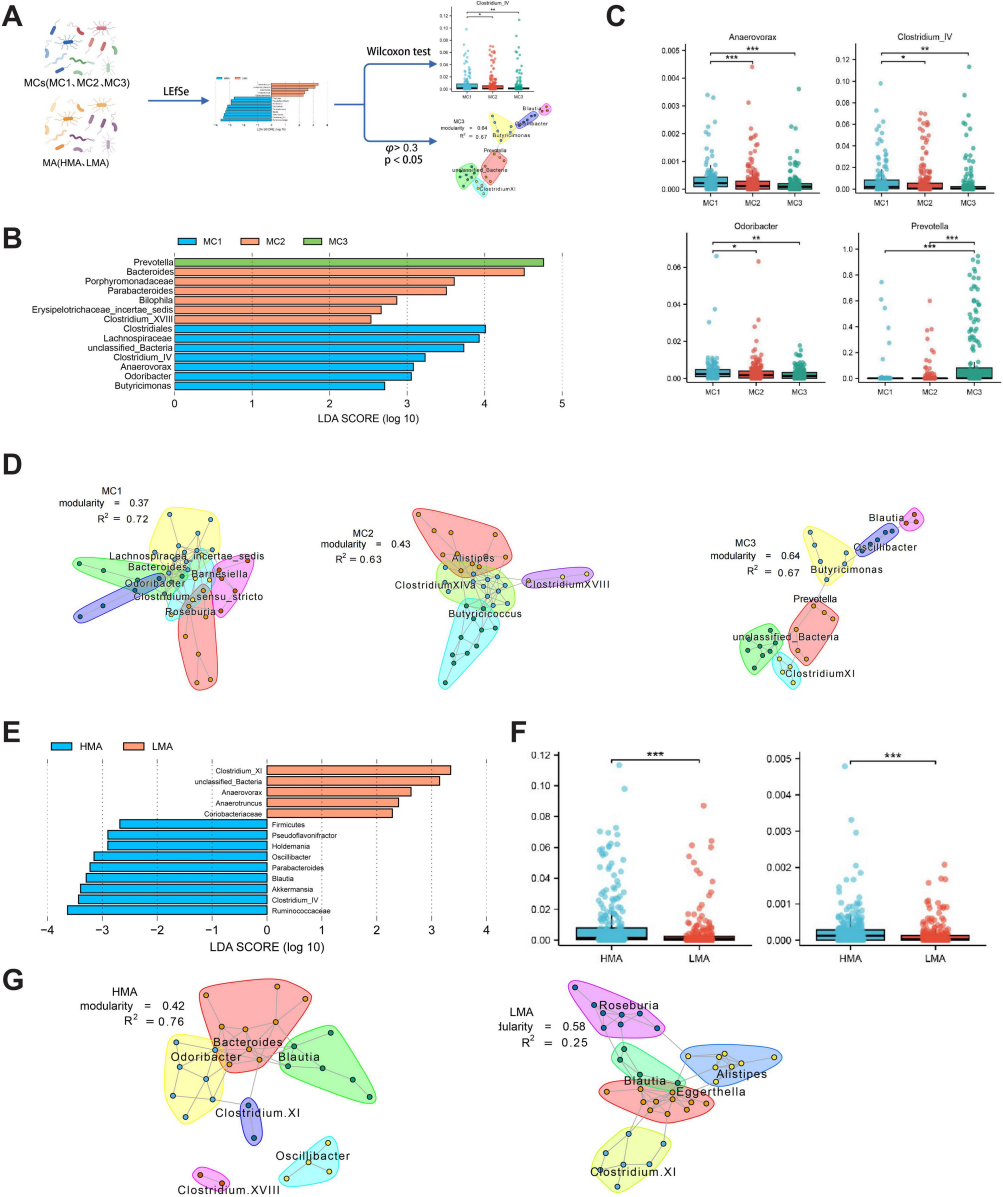
Gut microbial characteristics differences between metabolic clusters (MC1, MC2, and MC3) and microbial age groups (high microbial age and low microbial age). (A) Analysis workflow: Based on metabolic clusters and microbial age groups, linear discriminant analysis effect size analysis (linear discriminant analysis score>2, *P* value threshold*=*.05) and Wilcoxon tests were used to identify significantly different taxa, and co-occurrence network analysis was applied to reveal microbial community structure and functional characteristics. (B) Genera with statistically significant differences across metabolic subtypes (MC1-MC3) and microbial age groups (low microbial age and high microbial age) were identified using linear discriminant analysis effect size, with thresholds set at linear discriminant analysis score>2 and *P* value threshold=.05. (C) Box plots comparing the relative abundances of significantly different genera. (D) Genus coexpression networks: Nodes represent genera, node color indicates module membership, node size is proportional to mean abundance, and edge width reflects the strength of correlation (*φ*>0.3; *P* value threshold=.05). Modules were defined based on modularity maximization. (E) Linear discriminant analysis scores for microbial age groups. (F) Comparison of the abundance of significantly different taxa between high microbial age and low microbial age groups. (G) Co-occurrence network analysis for microbial age groups: microbial pairs were selected based on a φ coefficient>0.3 and *P* value threshold=.05; modules were defined using modularity maximization. Node color indicates functional modules, node size reflects relative abundance, and edge width indicates correlation strength. All significance tests were conducted using the Wilcoxon rank-sum test.

Using gut microbiome data from different metabolic clusters (MC1, MC2, and MC3) and MA groups (HMA and LMA), we used LEfSe analysis to identify bacterial genera that differed significantly between groups, with a threshold of LDA score>2 and *P* value threshold=.05. The differences in microbial abundance were further validated using the Wilcoxon test (Figure 5A).

To explore the species-level microbial differences within the MCs (MC1, MC2, and MC3), LEfSe analysis statistically delineated community variances, revealing genera with significant intergroup differences. Subsequently, we compared the abundance of these distinct genera across metabolic groups. The results indicated that Akkermansia, *Clostridium IV*, and Oscillibacter had significantly higher relative abundance in MC1 compared to other groups, while *Prevotella* was most abundant in MC3. MC1 was enriched with *Clostridium IV*, *anaerovorax, and odoribacter*, potentially linked to metabolic traits associated with glucose metabolism and renal function [29,30]. MC2 predominantly contained *Bacteroides, Parabacteroides, and Bilophila*, known for their roles in maintaining intestinal health and metabolic balance, reflecting MC2’s relatively healthy metabolic characteristics [31-33]. *Prevotella* was notably enriched in MC3, suggesting a close association with lipid metabolism disorders and potential cardiovascular risk [34]. These findings elucidate the differential distribution of microbial species across metabolic groups and their potential functional correlations (Figure 5B and C). The coexpression network characteristics and modular distribution of microbial species within the three MCs (MC1, MC2, and MC3) revealed microbial communities’ synergy and clustering traits under different metabolic states. In the MC1 group, the modularity value of the microbial coexpression network was 0.37 with an *R*² value of 0.72, indicating a less modular structure but still discernible clustering characteristics within this metabolic group. The primary modules included *Achromobacter aceae incertae sedis*, *Bacteroides*, and *Roseburia*. In MC2, the modularity value increased to 0.43 with an *R*² value of 0.63, suggesting clearer microbial community segregation. The main modules consisted of *Clostridium XIVa*, *Alistipes*, and *Butyricicoccus*, implying more distinct synergistic relationships among different microbes in MC2. The MC3 group exhibited the most pronounced modular features, with a modularity value of 0.64 and an *R*² value of 0.67, indicating a more independent and stable microbial network structure. The principal modules included *Butyricimonas*, *Prevotella*, and *Clostridium XI*. The high modularity value reflects a more differentiated microbial ecosystem within MC3, potentially linked to its characteristic metabolic state related to lipid metabolism abnormalities (Figure 5D).

We used LEfSe analysis to assess the statistical differences in microbiome composition between the HMA and the LMA and to compare the relative abundance of significantly different genera between these MA groups. The results indicated that in the HMA group, genera significantly differing from the LMA group included *Firmicutes*, *Pseudoflavonifractor*, *Holdemania*, *Oscillibacter*, *Parabacteroides*, *Blautia*, *Akkermansia*, *Clostridium IV*, and *Ruminococcaceae*, which may be linked to the metabolic features of HMA. In contrast, the LMA group was significantly enriched with *Clostridium XI, Anaerovorax, Anaerotruncus, and Coriobacteriaceae* genera. Notably, the relative abundance of *Clostridium IV* and *Pseudoflavonifractor* was significantly higher in the HMA group compared to the LMA group (*P*<.001), suggesting that HMA is associated with the enrichment of specific genera. These differences highlight the significant impact of MA on microbiome structure and function (Figure 5E and F). For the HMA group, the modularity value of the coexpression network was 0.42, with an *R*² value of 0.76, indicating a moderately modular microbial network with strong correlations. Key modules included *Bacteroides*, *Odoribacter*, *Blautia*, and *Clostridium XI*, suggesting high synergy and functional connections among these genera in the HMA group. In contrast, the LMA group exhibited a higher modularity value of 0.58 but a lower overall network correlation (*R*²=0.25). Major modules in the LMA group included *Roseburia*, *Blautia*, *Eggerthella*, and *Clostridium XI*, suggesting a more differentiated microbial distribution and the possible formation of independent ecological units. These differences reflect the varying effects of MA on microbial synergy and functional differentiation, with the HMA group exhibiting stronger metabolic functional co-ordination while the LMA group shows more differentiated ecological traits (Figure 5G).

### Association Between MA, MCs, and CVD Risk

**Figure 6.**
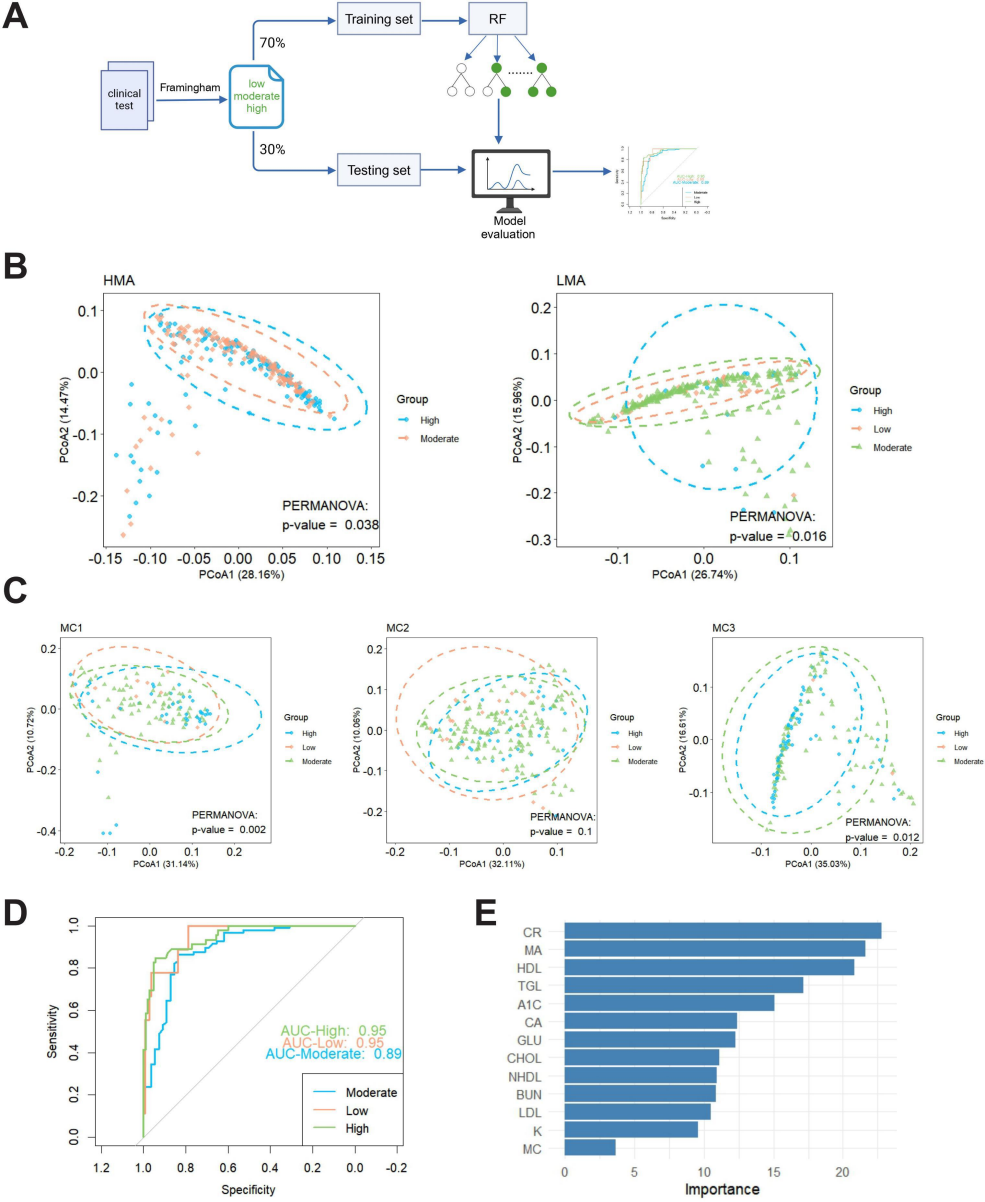
Cardiovascular disease risk stratification and gut microbiota analysis based on Framingham risk score. (A) Analysis workflow: participants were categorized into high-risk (green), medium-risk (blue), and low-risk (orange) groups based on the Framingham risk score. A cardiovascular risk prediction model was constructed using the random forest algorithm, incorporating microbial age, microbial clusters, and metabolic indicators, followed by microbiome distribution analysis. (B) Principal co-ordinates analysis of different risk groups in the high microbial age and low microbial age groups: shapes represent microbial age groups (circles for high microbial age and triangles for low microbial age), and colors represent risk levels (green: high risk, blue: medium risk, orange: low risk). (C) Principal co-ordinates analysis of cardiovascular risk groups in different metabolic clusters. In MC1 and MC3, shapes represent metabolic cluster categories, and colors indicate risk levels. (D) Receiver operating characteristic curve of the random forest model. Colors represent risk levels (orange: low risk, blue: medium risk, green: high risk). The area under the curve (AUC) is used to evaluate model performance, with AUC=1 indicating perfect prediction and AUC>0.9 indicating excellent prediction. (E) Importance ranking of variables in the RF model. The bars’ length indicates each variable’s contribution to the model’s predictive performance. A1C: hemoglobin A_1C_; BUN: blood urea nitrogen; CA: calcium; CHOL: cholesterol; CR: creatinine; GLU: glucose; HDL: high-density lipoprotein; K: potassium; LDL: low-density lipoprotein; MA: microbial age; MC: metabolic cluster; NHDL: non–high-density lipoprotein; TGL: triglyceride

Using the Framingham risk assessment, we evaluated the CVD risk of the study participants. Based on these risk scores (high, medium, and low), we analyzed differences in gut microbiome composition between the LMA and HMA groups. Principal Co-ordinates Analysis and permutational multivariate ANOVA were performed to reveal significant differences in microbiome composition across the different risk groups (Figure 6A). The results indicated that within both the HMA and LMA groups, significant differences in gut microbiome composition were observed across the high, medium, and low cardiovascular risk categories. Specifically, in the HMA group, there was a distinct difference in microbiome composition between the high-risk and medium-risk groups ( value threshold=.05), while in the LMA group, significant differences were found across all three risk categories (low, medium, and high) (*P* value threshold=.05; Figure 6B). Next, we examined the microbiome distribution across the different cardiovascular risk groups (high, medium, and low) within each MC. The results showed that in the MC1 group, significant differences in microbiome composition were observed between the high-risk and medium-risk groups in the HMA group (*P* value threshold=.05). In the MC2 group, no statistically significant differences were found between the risk groups (*P* value threshold=.05). In contrast, in the MC3 group, the high-risk and medium-risk groups exhibited significant differences in microbiome composition (*P* value threshold=.05). These findings suggest that microbiome composition significantly differs across cardiovascular risk groups, particularly between the high-risk and medium-risk categories, indicating a potential association between gut microbiome composition and CVD risk status (Figure 6C).

We further constructed a multifactorial prediction model using the RF algorithm, incorporating variables such as MA, MCs, and metabolic indicators to assess their predictive power for CVD risk. The model demonstrated a strong ability to differentiate between risk groups. Specifically, the curve for the medium-risk group (blue) was close to the top left corner, with an AUC of 0.89, indicating good predictive ability. The curve for the low-risk group (orange) showed an AUC of 0.95, indicating excellent discriminatory ability for this group. Similarly, the high-risk group (green) displayed an AUC of 0.95, comparable to the low-risk group, suggesting the model’s strong predictive power for both high and low-risk groups (Figure 6D). The importance of each variable in predicting CVD risk within the RF model was ranked. The results showed that creatinine had the highest importance score, indicating that CR contributed the most to the model’s predictive ability. MA and HDL were the next most influential variables, significantly impacting CVD risk prediction. In contrast, the importance of MCs was lower, contributing less to the model’s prediction. These findings suggest that the model primarily relies on key metabolic indicators (CR and HDL) and MA to predict CVD risk, with a relatively minor contribution from MCs (Figure 6E). These findings suggest that MA and MCs, as novel biomarkers, have not yet surpassed traditional indicators in predicting cardiovascular risk. Instead, they may serve as complementary factors to conventional markers or as tools for risk stratification in specific subpopulations. In the future, longitudinal data modeling, stratified analyses, or multimodal integration approaches should be employed to enhance their independent predictive power and explore their practical clinical value in informing early intervention strategies among high-risk populations.

## Discussion

### Principal Findings

This study reveals associations between different metabolic communities (MC1, MC2, and MC3) and gut microbiome composition while exploring the potential mechanistic links between the microbiome and metabolic health. LEfSe analysis identified key microbial taxa associated with specific metabolic features, emphasizing the complex interplay between host metabolism and gut microbiota.

MC1 is characterized by elevated glucose and BUN levels, suggesting potential insulin resistance, prediabetes, or diabetes. The microbiota in MC1 is significantly enriched with *Clostridium IV, Anaerovorax*, and *Odoribacter*, which may influence glucose metabolism through specific pathways. *Clostridium* species produce SCFAs, such as butyrate and acetate, critical in regulating insulin sensitivity [35,36]. Studies have shown that SCFAs regulate insulin sensitivity by activating G-protein coupled receptors, thereby influencing insulin secretion and glucose homeostasis. However, it should be noted that the association observed in this study between *Clostridium IV* and glucose metabolism indicators has not yet been experimentally validated for causality. Future investigations using fecal microbiota transplantation, germ-free animal models, or in vitro coculture experiments could help elucidate the specific mechanistic role of *Clostridium IV*, thereby enhancing the biological interpretability of the observed microbiome–metabolism associations. In addition, SCFAs can modulate the secretion of gut hormones that influence appetite and energy metabolism, further improving insulin sensitivity [37,38]. The metabolic imbalance observed in the MC1 group (elevated glucose) may involve gut dysbiosis (enrichment of *Clostridium IV*), impaired intestinal barrier function, and increased systemic inflammation, which together exacerbate insulin resistance and trigger glucose metabolism disorders.

In contrast, MC2 represents a metabolically healthy state, with normal glucose, lipids, and nitrogen metabolism markers. Key microbial taxa enriched in MC2, including *Bacteroides*, *Parabacteroides*, and *Bilophila*, likely contribute to maintaining metabolic homeostasis. *Bacteroides* play a pivotal role in the secondary metabolism of bile acids, influencing the gut-liver axis and lipid metabolism while reducing inflammation [39]. *Parabacteroides* may help maintain gut microbiota balance by inhibiting pathogenic bacterial growth, thereby supporting metabolic health [32]. The healthy metabolic state in MC2 (normal metabolic markers) is supported by a balanced microbiome composition (*Bacteroides* enrichment), which maintains normal bile acid metabolism and homeostasis of the gut-liver axis.

The MC3 group is characterized by elevated levels of cholesterol, LDL, and triglycerides, indicating lipid metabolism disorders and potential cardiovascular risk. Prevotella was significantly enriched in MC3, suggesting that these genera may aggravate lipid metabolic disturbances through multiple pathways. The *Prevotella* genus may promote systemic inflammation by producing lipopolysaccharide, inhibit reverse cholesterol transport, and contribute to atherosclerosis [40]. Lipid metabolism disorders in the MC3 group (elevated cholesterol and LDL) may be associated with gut dysbiosis (enrichment of *Prevotella*), increased inflammation, and oxidative stress, collectively heightening cardiovascular risk. It is important to note that the 16S ribosomal RNA (rRNA) sequencing technology used in this study is limited to genus-level resolution and cannot distinguish between functionally heterogeneous microbial species. For example, although *Prevotella* was enriched in the MC3 group, previous research has shown that different *Prevotella* species may have significantly distinct roles in lipid metabolism—some may promote lipopolysaccharide production and inflammation, while others may have anti-inflammatory potential. Therefore, while 16S rRNA sequencing is suitable for preliminary screening of microbial community structure, it has limitations for functional mechanism studies. Future research should use higher-throughput methods such as metagenomic sequencing or metabolomics to improve the depth and accuracy of functional characterization, which is particularly important for elucidating microbial mechanisms related to lipid metabolic disorders [41,42].

The microbiota enriched in the HMA group includes *Pseudoflavonifractor* and *Clostridium IV*. Studies indicate that *Pseudoflavonifractor* is a major producer of butyrate, which provides energy to the colonic mucosa and regulates host cell gene expression, inflammation, differentiation, and apoptosis [43]. *Clostridium IV* is also involved in butyrate production, a crucial metabolite for gut health that may influence energy metabolism and inflammatory responses [44]. This study observed significant associations between high MA and specific microbiome structures, enrichment of inflammation-related genera, and lipid metabolic abnormalities, suggesting a potential link between microbial aging and metabolic dysfunction. However, as this is a cross-sectional study, it is not possible to determine whether changes in MA temporally precede the onset of metabolic disorders, nor can causality be established. Thus, the proposed mechanistic explanations are based solely on correlation analyses and existing literature. Future studies should employ longitudinal follow-up or interventional designs to validate the temporal sequence and causal role of MA in the development of lipid disorders and cardiovascular risk.

Despite achieving meaningful results regarding the association between microbiological and metabolic characteristics in high-risk populations for diabetes, this study also has several limitations. First, the samples were collected during both infection and healthy phases. Although only healthy-phase samples were included in the primary analyses to minimize acute confounding, differences in physiological state may still have introduced potential biases in certain outcomes. We addressed this by applying data filtering, standardization, and sensitivity analyses to mitigate such biases as much as possible. Furthermore, although the study used large-scale iHMP data, the scope and sample size remain limited, potentially failing to capture the full diversity of high-risk diabetic populations globally. This limitation may be especially relevant when performing gender-stratified or subgroup analyses, which can result in reduced statistical power and an increased risk of type II error. Thus, some observed gender differences should be interpreted with caution, as they may be subject to bias or instability due to limited sample sizes. Future studies should aim to replicate and validate these findings using larger, multi-center datasets to strengthen the statistical reliability of subgroup analyses. Additionally, although the MA model offers a promising predictive tool, its accuracy requires further validation and optimization. The current MA model, based on 16S rRNA sequencing data, can identify genus-level microbial features but lacks the resolution to discern species-level functional heterogeneity or metabolic potential. This limits the model’s explanatory power regarding microbial functional status. Future research should integrate multi-omics functional data, such as metagenomic sequencing to identify microbial species and functional genes, as well as metatranscriptomics and metabolomics to measure gene expression and metabolic outputs, in order to more accurately capture microbial functional states and improve the construction of the MA model. Moreover, this study’s cross-sectional design prevents the establishment of causal relationships between MA and the risks of diabetes and cardiovascular diseases. Longitudinal studies are needed to explore the causal links and underlying mechanisms among these variables. Finally, although the study used the iHMP platform, which provided relatively comprehensive clinical and microbiome data, several key lifestyle variables were either missing or inadequately documented. These variables include dietary intake, medication use (such as antidiabetic agents and statins), physical activity, and smoking status. As these factors are widely recognized to significantly influence gut microbiota and metabolic status, their absence may confound the observed associations between MA, metabolic subtypes, and cardiovascular risk.

Therefore, future research could advance in several key areas. On the one hand, given the complex interactions between metabolic dysregulation and the microbiota in diabetes and CVD, researchers could explore the functional differences of microbiota across different metabolic subtypes and their association with disease progression. By integrating high-throughput sequencing technologies and metabolomics, researchers could further elucidate how gut microbiota influences high-risk diabetic populations. On the other hand, with the ongoing advancement of artificial intelligence and machine learning technologies, future studies could enhance the predictive capability of MA about metabolic disorders and cardiovascular risk through multidimensional data integration. For instance, deep learning models based on multiomics data could more accurately identify potential biomarkers and assist in developing personalized clinical interventions. Furthermore, future interventional clinical trials will help verify the practical value of MA in the early prevention of diabetes and CVDs, thus advancing the clinical translation of precision medicine.

### Conclusion

This study reveals significant features of MCs and MA in individuals at high risk for diabetes, exploring the complex relationships between these features, gut microbiota, metabolic indicators, and CVD risk (Graphic abstract). Using k-means clustering, we classified high-risk individuals into three distinct metabolic subtypes, each associated with glucose metabolism disturbances, metabolic homeostasis, and lipid metabolism abnormalities. RDA identified age as the primary covariate influencing gut microbiome composition, with significant differences observed between the microbiomes of the different metabolic subtypes. An RF model was employed to predict the relationship between MA and CVD risk, revealing a significant predictive correlation between MA and chronological age. LEfSe analysis further identified microbial taxa with significant differences across metabolic subtypes and MA groups. Ultimately, we found that the HMA group exhibited stronger lipid metabolism disturbances and CVD risk characteristics, suggesting that MA may serve as a potential biomarker for early assessment and intervention in high-risk diabetic populations.

**Figure 7.**
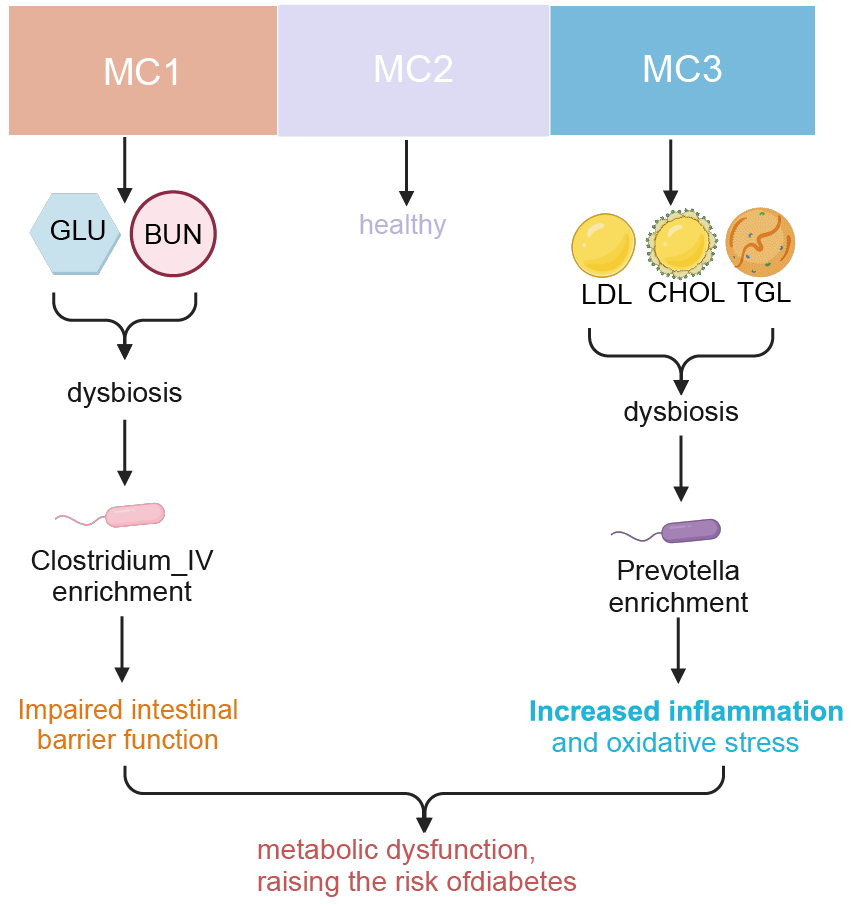
Mechanistic diagram of the association between metabolic clusters, gut microbiota, and diabetes risk. MC1 and MC3 are characterized by impaired glucose and lipid metabolism, respectively. They are accompanied by gut microbiota dysbiosis enriched with *Clostridium IV* and *Prevotella*, which leads to compromised intestinal barrier function and increased inflammation and oxidative stress. BUN: blood urea nitrogen; CHOL: cholesterol; GLU: glucose; LDL: low-density lipoprotein; MC: metabolic cluster; TGL: triglyceride.

The main contribution of this study lies in its systematic identification of the associations between MCs and MA, highlighting how these factors jointly influence metabolic disturbances and CVD risk in high-risk diabetes populations. This finding not only provides new insights into the complex pathophysiological mechanisms underlying diabetes and its complications but also offers a novel biological marker for personalized treatment and early intervention in high-risk diabetes individuals. As a potential predictor of metabolic dysfunction and CVD risk, MA holds significant clinical relevance. It may not only assist in predicting the onset of diabetes and CVD but also provide a new biological foundation for early intervention in high-risk groups. Thus, this study offers clinicians more precise risk assessment tools, facilitates the development of personalized medicine, and introduces new strategies for preventing and treating diabetes and CVDs (Figure 7).

## Supplementary material

10.2196/73119 Multimedia Appendix 1 Baseline demographic and metabolic characteristics of subjects included from the iHMP and exercise subproject cohorts.

## Abbreviations

AUC: area under the curve
BUN: blood urea nitrogen
CVD: cardiovascular disease
dbRDA: distance-based redundancy analysis
HDL: high-density lipoprotein
HMA: high microbial age
iHMP: Integrative Human Microbiome Project
LDA: linear discriminant analysis
LDL: low-density lipoprotein
LEfSe: linear discriminant analysis effect size
LMA: low microbial age
MA: microbial age
MC: metabolic cluster
RDA: redundancy analysis
RF: random forest
rRNA: ribosomal RNA
SCFA: short-chain fatty acid
